# A Low-Cost Colorimetric Assay for the Analytical Determination of Copper Ions with Consumer Electronic Imaging Devices in Natural Water Samples

**DOI:** 10.3390/molecules28124831

**Published:** 2023-06-17

**Authors:** Argyro G. Gkouliamtzi, Vasiliki C. Tsaftari, Maria Tarara, George Z. Tsogas

**Affiliations:** Laboratory of Analytical Chemistry, School of Chemistry, Faculty of Sciences, Aristotle University of Thessaloniki, GR-54124 Thessaloniki, Greece

**Keywords:** colorimetric determination, DDTC complexation, copper ions, simple imaging devices, natural water samples

## Abstract

This study reports a new approach for the determination of copper ions in water samples that exploits the complexation reaction with diethyldithiocarbamate (DDTC) and uses widely available imaging devices (i.e., flatbed scanners or smartphones) as detectors. Specifically, the proposed approach is based on the ability of DDTC to bind to copper ions and form a stable Cu-DDTC complex with a distinctive yellow color detected with the camera of a smartphone in a 96-well plate. The color intensity of the formed complex is linearly proportional to the concentration of copper ions, resulting in its accurate colorimetric determination. The proposed analytical procedure for the determination of Cu^2+^ was easy to perform, rapid, and applicable with inexpensive and commercially available materials and reagents. Many parameters related to such an analytical determination were optimized, and a study of interfering ions present in the water samples was also carried out. Additionally, even low copper levels could be noticed by the naked eye. The assay performed was successfully applied to the determination of Cu^2+^ in river, tap, and bottled water samples with detection limits as low as 1.4 µM, good recoveries (89.0–109.6%), adequate reproducibility (0.6–6.1%), and high selectivity over other ions present in the water samples.

## 1. Introduction

Copper (Cu) is a metal that belongs in group 11 of the periodic table, and it is found naturally in the earth’s solid crust. It is a soft, malleable, and ductile metal with great chemical and physical properties such as remarkably high thermal and electrical conductivity. It is such an important metal that it gave its name to an era of the prehistoric ages, “the copper age”. For centuries, humans have used it to produce copper alloys including brass and bronze [1]. Copper is one of the most common metals used to manufacture many products, including electrical and electronics equipment; building construction, machinery, and consumer products; and spare parts for hydraulic systems and plumbing materials such as household water pipes [2,3]. It can be found in drinking water, when the water passes through domestic plumbing that contains copper piping or fittings, in its most stable oxidation state of Cu^2+^. In many countries worldwide, the monitoring of copper in tap water systems [4] and urban wastewater [5] and the possible health effects of its concentration is of great importance.

The human body needs an amount of copper as an essential micronutrient to stay healthy, but in high concentrations, it is considered to be harmful. According to the Food and Drug Administration, a small daily dosage of copper of 1.4 mg for men and 1.1 mg for women is essential to maintain good human health [6]. Major food sources of copper are beef liver, shellfish, nuts, vegetables, mushrooms, and chocolate [7]. Exposure to high daily doses of copper or metal accumulation can cause serious health problems, including Wilson’s disease [8]. Short-term exposure to high levels of copper can cause gastrointestinal distress, and long-term exposure and severe cases of copper poisoning may cause anemia, disruption of liver and kidney functions, and finally cell death—a recently studied disease called cuproptosis [9,10]. Therefore, the development of rapid, accurate, and easily applicable methods for the quantitative determination of copper ions in water samples is considered of great importance.

In terms of analytical chemistry, a vast number of recent analytical methods have been developed and validated for the quantification of Cu^2+^ in several samples, including biochemical, food, water, and wastewater samples. These methods are mainly spectroscopic and require expensive instrumentation and specialized staff, including batch UV-Vis spectrometry [11,12,13,14], flame atomic absorption spectroscopy (FAAS) [15,16,17,18], graphite furnace atomic absorption spectrometry (GFAAS) [19,20], inductively coupled plasma (ICP) [21,22], flow injection [23], and fluorescence [24,25,26,27].

Diethyldithiocarbamate (DDTC) is a dithiocarbamate pesticide and has been used for the complexation of many metal ions such as Cr^3+^, Zn^2+^, Ni^2+^, Co^2+^, Fe^3+^, and Mn^2+^, resulting in the formation of stable metal complexes [28]. One of the various metal ions that interacts strongly with DDTC is Cu^2+^, forming a stable Cu(II)-DDTC complex [29,30,31,32].

Although the aforementioned analytical methods are accurate, provide low detection limits, and are reproducible, they, unfortunately, require trained personnel, expensive instrumentation and consumables, and high solvent volumes for the analytical detection of copper ions. In remote areas or in facilities with reduced budgets, these drawbacks are major obstacles to the development of precise analytical methods when multiple samples must be analyzed in short time periods. Motivated by these problems, we developed a new colorimetric analytical method for the determination of Cu^2+^ in natural water samples. Our research team has taken a step forward in the creation and development of low-cost and simple analytical colorimetric sensors, which can be applied with minimal resources and instrumentation to provide rapid and reliable results in the determination of various analytes carried out by a smartphone detector or a flatbed scanner [33,34,35].

In an effort to further simplify the overall analytical process, we report herein a 96-well plate assay for the analytical determination of Cu^2+^ relying on the colorimetric alteration that is analogous to copper concentration because of the complexation reaction between copper cations and DDTC molecules in a neutral environment. The analytical method designed is cheap, easy, and fast to implement, and the analytical protocol used for this determination is easily feasible with minimal technical expertise and is instrument-free. Based on this protocol, the determination of copper ions can be performed by the sequential addition of the pH-conditioning reagent (distilled water or the necessary acid or base), the complexation reagent (DDTC), and the analyte in the 96-well plate cells. We waited for a short time period at room temperature for the full development of the complex color, taking a photograph with a smartphone camera or a flatbed scanner and measurement with the ImageJ program. The color change during the development of this method was also evident with the naked eye (concentrations up to 10 μM had a pale yellow color, concentrations from 10 to 25 μM had a relatively bright yellow color, and concentrations above 25 μM had a very intense yellow color). These three areas can be distinguished by an appropriate experimental design and a semi-quantitative determination of copper ions can be accomplished. Finally, this method’s applicability was tested for the determination of Cu^2+^ with promising results in terms of accuracy, sensitivity, and reproducibility.

## 2. Results and Discussion

### 2.1. Optimization Parameters

The possible effect of many parameters for the optimization of the proposed analytical procedure for the colorimetric determination of Cu^2+^ in natural water samples was studied in detail.

#### 2.1.1. Effect of Reagent Series

**Figure 1:**
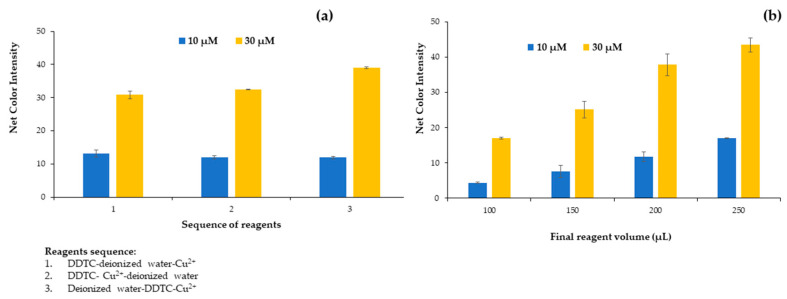
Effect of (**a**) reagent sequence and (**b**) final reagent volume on the net color signal for the proposed method.

After the preliminary experiments for the successful formation of the colored complex of Cu^2+^ and DDTC, the first parameter studied was the sequence of reagents added to the cells of the plate. We tested three different approaches for the addition of the reagents to complex the copper ions and thus to form the colored complex. On the first try, we added DDTC–deionized water–Cu^2+^ (1), on the second try DDTC–Cu^2+^–deionized water (2), and on the third try deionized water–DDTC–Cu^2+^ (3) for 10 and 30 μmol L^−1^ Cu^2+^ concentrations. From Figure 1a, it is obvious that at the lower concentration, the sequence of the reagents had no effect on the net color intensity (the color of the blank minus color of the sample), but at the higher concentration, a significant increase in the net color intensity was observed for the third reagent sequence. This is expected from the point of view of analytical chemistry because it is optimal for the analyte to be added last, which occurs in the analysis of real samples. Thus, the third sequence of reagent addition was used for the experiments.

#### 2.1.2. Effect of Plate Volume

The next parameter studied was the influence of the final reagent volume contained in the plate cells for 10 and 30 μmol L^−1^ of Cu^2+^. The plate cells had a maximum volume content of 250 μL, so 4 different volumes were tested from 100 to 250 μL. Volumes less than 100 μL were difficult to handle with minimum errors, and taking a photograph was impossible to accomplish. As depicted in Figure 1b, there is an upward trend of the signal up to the maximum possible volume that can be placed on the plate. The main disadvantage of using smaller volumes was the difficulty of taking the photo and correspondingly measuring the color variation due to the limited surface area visible to the smartphone lens because the solution was at a low level inside each plate cell.

#### 2.1.3. Effect of DDTC Concentration

**Figure 2:**
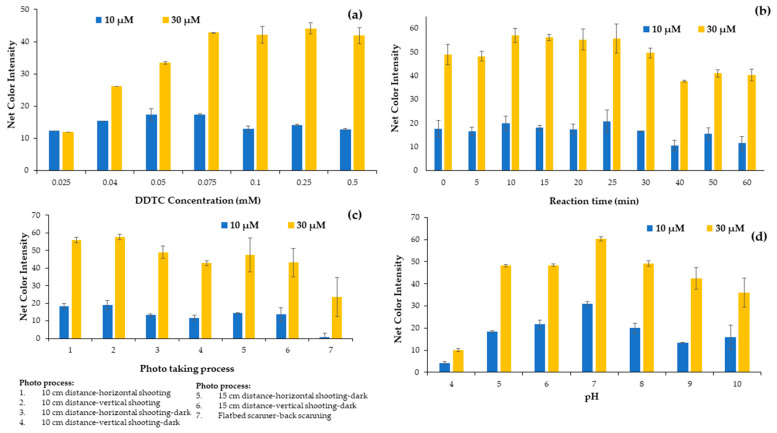
Effect of (**a**) DDTC concentration, (**b**) reaction time, (**c**) photo process and (**d**) pH on the net color signal for the proposed method.

After determining the reagent sequence and the final volume added in each cell of the plate that we used in the analytical methodology, the influence of DDTC concentration in the evolution of the complexation reaction was studied in the range from 0.025 to 0.5 mmol L^−1^ by adding the appropriate diluted solution to the well. The DDTC concentrations of the solutions studied were 0.025, 0.04, 0.05, 0.075, 0.1, 0.25, and 0.5 mM. The experiments proved that maximum colorimetric values were achieved for a DDTC concentration of 0.25 mmol L^−1^. Thus, this concentration was chosen for the subsequent experiments (Figure 2a). It is obvious that for lower concentrations there were not enough DDTC molecules to complex with Cu^2+^, while for higher concentrations a flattening of the net colorimetric signal was observed. Even though the net signal was similar for DDTC concentrations higher than 0.075 mM, to ensure that Cu^2+^ quantitatively reacted with DDTC the 0.25 mmol L^−1^ DDTC concentration value was chosen for the experiments.

#### 2.1.4. Effect of Reaction Time

The complexation reaction between copper ions and DDTC molecules is rapid even at room temperature, and its intensity can be measured immediately after the addition of the DDTC molecules. Nevertheless, due to the quantitative formation of the complex during this study, it was necessary to study the reaction time for the colorimetric determination of the copper ions. The influence of the reaction time was studied in the range of 0 to 60 min with time intervals of 5 min from 0 to 30 min and intervals of 10 min from 30 to 60 min of the reaction time, as shown in Figure 2b. The slight decrease in the colorimetric signal for the higher internal times was attributed to the quenching of the color after a period of time. It is obvious from Figure 2b that a reaction time of 10 min is adequate for taking the photograph.

#### 2.1.5. Effect of Photo Capture Apparatus

Next, the selection of the apparatus used to capture the photographs of the plates was studied. A smartphone and a flatbed scanner were used for this purpose. Because, unfortunately, in our lab there is a scanner that scans only from the bottom, we had the one and only option of taking the photo from the bottom of the plate, with disappointing results compared to the smartphone used. The smartphone photograph was tested with different layouts and alignments at a 10 cm distance from the plate, and we used a dark protective box to identify the effect of environmental conditions on the photo taking and consequently on the alarm signal (Figure 2c). The net signal intensity (sample minus blank signal) decreased when measured with the flatbed scanner, and although the scanner is considered to be less affected by external factors such as solar radiation, we had to rely on the mobile phone due to the large signal difference (Figure 2c).

#### 2.1.6. pH Effect

DDTC molecules and Cu^2+^ ions reacted quickly for the generation of a yellow Cu(DDTC)_2_ complex. It has been proved in previous reports that this complexation reaction takes place at neutral pH values [31,32]. In contrast, the chelation procedure of DDTC and Cu^2+^ was found in previous reports to be sensitive to pH [36], and the pH value of the solutions or the water samples might affect the efficiency of the proposed method. Motivated by these findings, we decided that the next parameter we should study in detail was the influence of pH on the formation of the metal complex. We tested the effect of different concentrations of hydrochloric acid and sodium hydroxide; in order to achieve higher colorimetric values, pH values from 4 to 10 were studied. Dilute HCl and NaOH solutions were prepared and used to achieve different pH values. As shown in Figure 2d, the color signal of the Cu(DDTC)_2_ complex reached a peak as the pH raised from 4 to 7, but the color intensity decreased as the pH increased to 10. The maximum color intensity was determined at pH 7, which was conducive to the effective formation of the complex. For possible highly acidic or basic samples, buffer solutions such as phosphate buffer can replace deionized water and regulate the pH at 7, without interfering in the DDTC-Cu complexation reaction. When the pH exceeded 7, the hydrolysis of Cu^2+^ played an important role in the solution. In acidic pH, the DDTC is unstable [37], because Na-DDTC decomposed to release the free amine and carbon disulfide. Therefore, a pH of 7 was chosen for the experimental process.

#### 2.1.7. Effect of Ionic Strength

**Figure 3:**
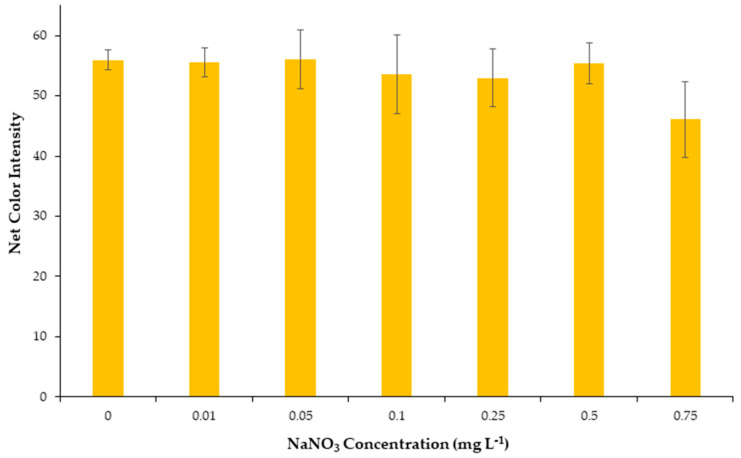
Effect of salinity on the net color signal for the proposed method.

Ionic strength occasionally affects the formation, the stability of metal complexes, and the robustness of analytical methods. Therefore, the effect of ionic strength was considered as an important parameter to study and carried out by the addition of different concentrations of NaNO_3_ solutions to the well cells in the range between 0.01 and 0.75 mol L^−1^. As can be seen from Figure 3, the alteration of ionic strength had practically no influence on the stability of the Cu-DDTC complex and consequently on the net colorimetric signal in the range between 0.01 and 0.75 mol L^−1^, which is in agreement with previous reports [38]. Additionally, these findings show that the proposed method is suitable for the detection of copper ions in samples with high ionic strength, such as seawater samples, and also prove the robustness of the method to important changes such as the effect of salinity. In conclusion, it was chosen that no salt would be added in the next experiments.

### 2.2. Method Validation

Regarding the validation of the method in terms of analytical chemistry, the proposed colorimetric method was studied in terms of accuracy, linearity, limits of detection (LOD) and quantification (LOQ), precision, and selectivity.

#### 2.2.1. Linearity, Precision, and Limits of Detection (LOD) and Quantification (LOQ)

**Figure 4:**
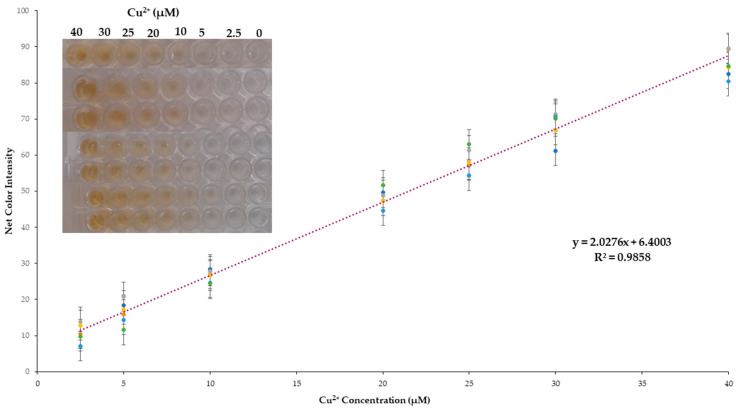
Cumulative calibration curve for the proposed method.

The developed method showed adequate linearity of Cu^2+^ concentrations in the range between 2.5 and 40 μmol L^−1^ (Figure 4). At higher concentrations, color saturation occurred, and a plateau was reached. The values of the color signal were almost identical for concentrations above 40 μM due to the color saturation. The regression equation was obtained by integrating the results from 49 standard solutions measured on different working days (n = 7) for the validation of the method. In this way, the calibration curve is far more representational, including possible variations from day-to-day measurements, and the regression equation obtained was:

CI = 2.03 (±0.04) [Cu^2+^] + 6.40 (±0.88), with R^2^ = 0.986

where CI is the color intensity measured by the method.

The within-day precision (intra-day) and between-days precision (inter-day) were validated at copper concentrations of 10 and 30 μmol L^−1^ by repetitive measurements of different samples (n = 7) on 7 different days. The intra-day relative standard deviation obtained (RSD) was 2.3% and 0.6%, while the inter-day was 6.1% and 3.7% for 10 and 30 μmol L^−1^ Cu^2+^, respectively.

Finally, the limit of detection (LOD) and limit of quantification (LOQ) were calculated as LOD = 3.3 × SDi/s and LOQ = 10 × SDi/s, where SDi is the standard deviation of the intercept, and s is the slope of all regression lines. The LOD/LOQ calculated for the determination of Cu^2+^ was 1.4 and 4.3 μmol L^−1^, respectively.

#### 2.2.2. Interference Study Selectivity

**Figure 5:**
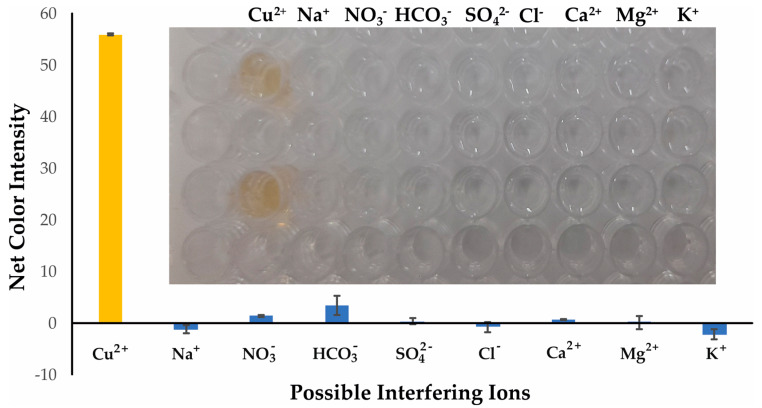
Selectivity of Cu^2+^ determination under the optimum experimental conditions for 30 μmol L^−1^. Error bars are the standard deviation for n = 3.

**Figure 6:**
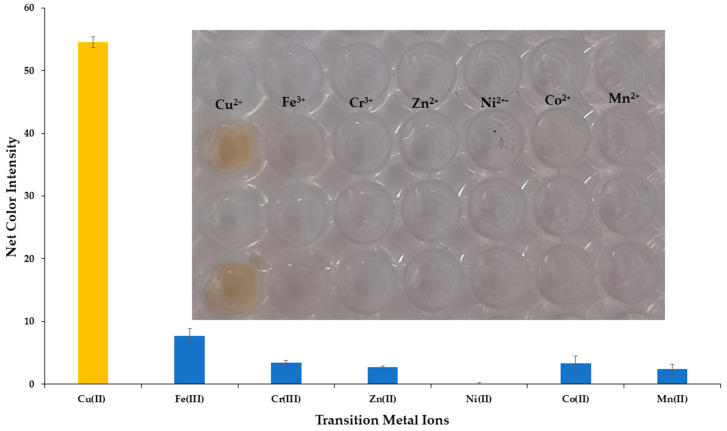
Selectivity of Cu^2+^ determination versus transition metal ions under the optimum experimental conditions for 30 μmol L^−1^. Error bars are the standard deviation for n = 3.

The selectivity of the developed colorimetric method was validated against representative cations and anions that are the most frequently encountered and in higher concentrations in the natural water samples. All potential interferents were analyzed at different concentrations representative of their existence in natural surface waters and are shown in Table 1, whereas Cu^2+^ was at 30 μmol L^−1^. The influence of the anions and cations is depicted in Figure 5. None of the selected ions formed the DDTC complex in the specific experimental conditions, and this could be observed even by the naked eye, as shown in the integrated photo of Figure 5. We also studied the selectivity of our method towards the transition metal ions. As expected, Cr^3+^, Zn^2+^, Mn^2+^, and Ni^2+^ did not produce a colored complex with DDTC [29], and thus no interference was detected for concentrations up to 20 mg L^−1^ for each metal. Additionally, for Fe^3+^ and Co^2+^ a pale yellow color was formed at concentrations of 10 mg L^−1^, while for concentrations up to 2.0 mg L^−1^ no colored complex was achieved for each metal (Figure 6 and integrated photo). This concentration is 10 times higher than that permitted by the European Union (0.2 mg L^−1^), so no interference can be anticipated in surface water and drinking water samples [39]. It is clear that none of the most abundant ions in natural waters has a significant influence on the analytical signal of the proposed method, and as a result the method can be characterized as selective for the analytical determination of copper ions.

### 2.3. Real Sample Applicability

Seven real samples—four bottled water samples from northern and northwestern Greece; two river water samples from the region of Epirus (northwestern Greece); and tap water from the city center of Thessaloniki—were measured with the developed colorimetric method. The samples were handled as described in the Experimental Section, and the results are presented in Table 2. The recoveries of the determined spiked levels of Cu^2+^ were satisfactory and ranged between 89.0 and 109.6%, with a good standard deviation.

## 3. Materials and Methods

### 3.1. Reagents and Solutions

Copper sulfate pentahydrate (CuSO_4_ × 5H_2_O) was purchased from Penta chemicals unlimited (Prague, Czech Republic). Sodium chloride, sodium sulfate anhydrous, hydrochloric acid, and sodium hydrogen carbonate were provided by Merck (Darmstadt, Germany). Magnesium nitrate hexahydrate and sodium nitrate were provided by Panreac (Madrid, Spain), while calcium nitrate tetrahydrate and potassium nitrate were bought from Chem Lab NV (Zedelgem, Belgium). Diethyldithiocarbamate sodium salt (Na-DDTC, C_5_H_10_NNaS_2_ × 3H_2_O) was provided by Carl Roth GmbH (Karlsruhe, Germany). For the selectivity of the proposed method towards transition metal ions (Fe^3+^, Ni^2+^, Zn^2+^, Cr^3+^, Co^2+^, and Mn^2+^), 6 salts were used, and stock solutions of 100 mg L^−1^ were prepared. Nickel (II) sulfate hexahydrate ΝiSO_4_ × 6H_2_O was purchased from Penta chemicals unlimited (Prague, Czech Republic). Chromium (III) nitrate nonahydrate Cr(NO_3_)_3_ × 9H_2_O, Zinc sulfate heptahydrate ZnSO_4_ × 7H_2_O, and Cobalt (II) nitrate hexahydrate Co(NO_3_)_2_ × 6H_2_O were provided by Merck (Darmstadt, Germany). Iron (III) nitrate nonahydrate Fe(NO_3_)_3_ × 9H_2_O was bought from Chem Lab NV (Zedelgem, Belgium), and finally Manganous chloride tetrahydrate MnCl_2_ × 4H_2_O was purchased from BDH Chemicals (Poole, England).

All chemical substances were of analytical grade, and all the solutions were prepared with deionized water. The standard stock Cu^2+^ solution (1.0 mmol L^−1^) was prepared weekly in deionized water, while the standard stock DDTC solution (1.0 mmol L^−1^) was prepared daily in deionized water. Working solutions of Cu^2+^ and DDTC were prepared every day by diluting the stock solutions in deionized water. Standard stock solutions of 2.0 mol L^−1^ HCl and NaOH were used for the pH study. Additionally, for the selectivity study, different cation and anion stock solutions of 1000 mg L^−1^ for each ion were prepared in deionized water. Finally, sodium nitrate stock solution (2.0 mol L^−1^) used for the salinity study was prepared by dissolving the appropriate amount in deionized water.

### 3.2. Apparatus

The instrumentation used for this study is clearly minimal. The pH values of the tested acidic and alkaline solutions were measured with a pH meter (Orion). The images of the devices were captured at specific time periods and at the same distance and angle of the photograph taken, using a mobile smartphone (Samsung Galaxy A21s, Samsung, Ridgefield Park, NJ, USA) or a common flatbed scanner (HP Scanjet 4850, Hewlett Packard Inc., Palo Alto, CA, USA).

### 3.3. Experimental Process

**Figure 7:**
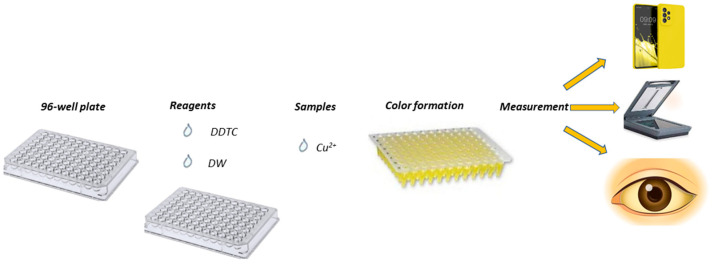
Experimental procedure of the proposed colorimetric method.

The experimental process was easy to perform, with absolutely no demand for laboratory instrumentation. In brief, a few μL of deionized water (pH = 7), DDTC (62.5 μL, 0.25 mmol L^−1^), and the appropriate volumes (e.g., 75 μL for 30 μmol L^−1^ Cu^2+^) of sample/standard solutions were sequentially added in the wells of the plate for a final volume of 250 μL. For the blank samples, deionized water was used, replacing copper ions. After a short time of 10 min, at room temperature, a photograph of the plate was taken. The photographs taken after this analytical procedure were saved as JPEG files (minimum analysis of 300 dpi), and the ImageJ program (Version 1.53.k) in RGB mode in the blue area was used for the measurement of the mean color intensity (Figure 7). From the ImageJ software, the blue type of images were chosen, and an elliptical shape was selected with the same area in every measurement carried out. The mean color intensity was determined, and the results were transferred to Excel and formed into the figures presented herein.

### 3.4. Real Samples

The method was tested on seven different natural water samples. The origin of the samples was divided into three distinct categories: river samples, bottled samples, and tap water samples. Four bottled water samples were purchased from local stores in the Thessaloniki city center. Two river water samples were collected in the Epirus region (NW Greece), from the Louros and Acheron rivers, stored in glass containers of 500 mL each, and filtered through a Whatman 0.45 μm filter to remove any suspended solids. Finally, the tap water sample was collected from the water supply system of the Aristotle University of Thessaloniki campus. All samples were stored at 4 °C in the refrigerator.

## 4. Conclusions

A fast, equipment-free, and reliable colorimetric method for the analytical determination of Cu^2+^ in natural water samples was developed and validated. The developed sensor utilized only two readily available reagents and is desirable for real-time applications. The developed analytical method relied on the colorimetric differences generated at neutral conditions by the different copper ion concentrations, and the color intensity was recorded by a domestic smartphone camera. The calibration curve obtained was linearly proportional to the concentration of the analyte. The maximum allowable concentration in surface waters according to Directive (EU) 2020/2184 of the European Parliament and of the Council on the quality of water intended for human consumption is 2.0 mg/L [39]. Thus, in μM concentration units, this corresponds to 31.5 μM, which is much higher than the limit of detection accomplished by this method. Furthermore, the analytical methodology developed is robust, can analyze many samples in a short time period (up to 96 different samples), and permits the analysis of Cu^2+^ at low μM levels (LOD: 1.4 μmol L^−1^). The levels of Cu^2+^ in the spiked analyzed real samples were within acceptable recovery limits from 89.0 to 109.6%.

## Figures and Tables

**Table 1:** Interfering ion concentrations used for the selectivity study.

Interfering Cations	Concentration (mg L^−1^)	Interfering Anions	Concentration (mg L^−1^)
Na^+^	50	NO_3_^−^	50
Ca^2+^	100	HCO_3_^−^	500
Mg^2+^	100	SO_4_^2−^	25
K^+^	25	Cl^−^	100

**Table 2:** Applicability of the colorimetric method for Cu^2+^ analysis in water samples.

Samples	Spiked (μmol L^−1^)	Found (μmol L^−1^)	% Recovery (±RSD, n = 5)
Bottled water 1	10 30	10.1 28.6	101.5 ± 0.3 95.2 ± 1.5
Bottled water 2	10 30	10.8 29.1	107.6 ± 6.3 96.9 ± 3.5
Bottled water 3	10 30	10.3 29.1	102.8 ± 6.5 96.9 ± 3.2
Bottled water 4	10 30	8.9 27.0	89.0 ± 5.0 89.9 ± 5.8
River water 1	10 30	11.0 28.7	109.6 ± 7.1 95.6 ± 4.6
River water 2	10 30	9.9 29.4	98.8 ± 8.4 97.8 ± 5.0
Tap water	10 30	9.2 28.1	92.3 ± 4.7 93.6 ± 0.6

## Data Availability

Not applicable.

## Sample Availability

DDTC, Cu2+ and all other compounds used in this work are available from the authors.
